# Supplementary material for: Structural insights into the mechanism and dynamics of proteorhodopsin biogenesis and retinal scavenging
Source: Nat Commun. 2024 Aug 13;15:6950. doi: 10.1038/s41467-024-50960-3 (PMC11322631; doi:10.1038/s41467-024-50960-3)
Supplement: Supplementary file 1 — Supplementary Information [file 41467_2024_50960_MOESM1_ESM.pdf]

Supplementary Information

Structural insights into the mechanism and dynamics of proteorhodopsin biogenesis and retinal scavenging

Stephan Hirschi[1,+,*], Thomas Lemmin[1,*], Nooraldeen Ayoub[1], David Kalbermatter[1], Daniele Pellegata[1], Zöhre Ucurum[1], Jürg Gertsch[1] & Dimitrios Fotiadis[1,*]

[1]Institute of Biochemistry and Molecular Medicine, University of Bern, 3012 Bern, Switzerland

[+]Present address: Department of Biochemistry, University of Oxford, OX1 3QU Oxford, United Kingdom

[*]Correspondence should be addressed to S.H. (email: stephan.hirschi@bioch.ox.ac.uk), T.L (email: thomas.lemmin@unibe.ch) or D.F. (email: dimitrios.fotiadis@unibe.ch)

Supplementary Figures

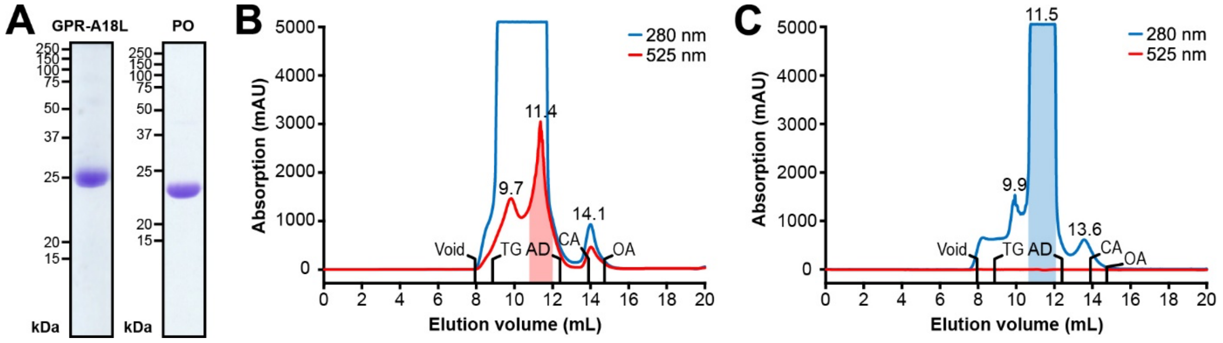

Supplementary Fig. 1: Purification of GPR-A18L and proteoopsin (PO). (A) SDS-PAGE analysis of purified GPR-A18L and PO reveals highly pure and homogeneous samples. Notably, both proteins run as single bands, with GPR-A18L at around 25 kDa and PO at around 23 kDa. Size-exclusion chromatograms of GPR-A18L (B) and PO (C) on a Superdex 200 Increase 10/300 column with absorption at 280 nm and 525 nm (absorption maximum of retinal Schiff base). Main peaks at around 11.4 mL and 11.5 mL correspond to oligomeric protein and peaks at around 14.1 mL and 13.6 mL to monomers as observed previously (elution volume of pentameric GPR was 11.3 mL).[1,2] Both elution profiles also indicate the presence of higher molecular weight aggregates (9.7 and 9.9 mL). Fractions containing oligomeric protein used for cryo-EM are highlighted in red (GPR-A18L) or blue (PO). The void and elution volumes of molecular weight markers are indicated: Thyroglobulin (TG, 669 kDa), aldolase (AD, 158 kDa), conalbumin (CA, 75 kDa) and ovalbumin (OA, 43 kDa).

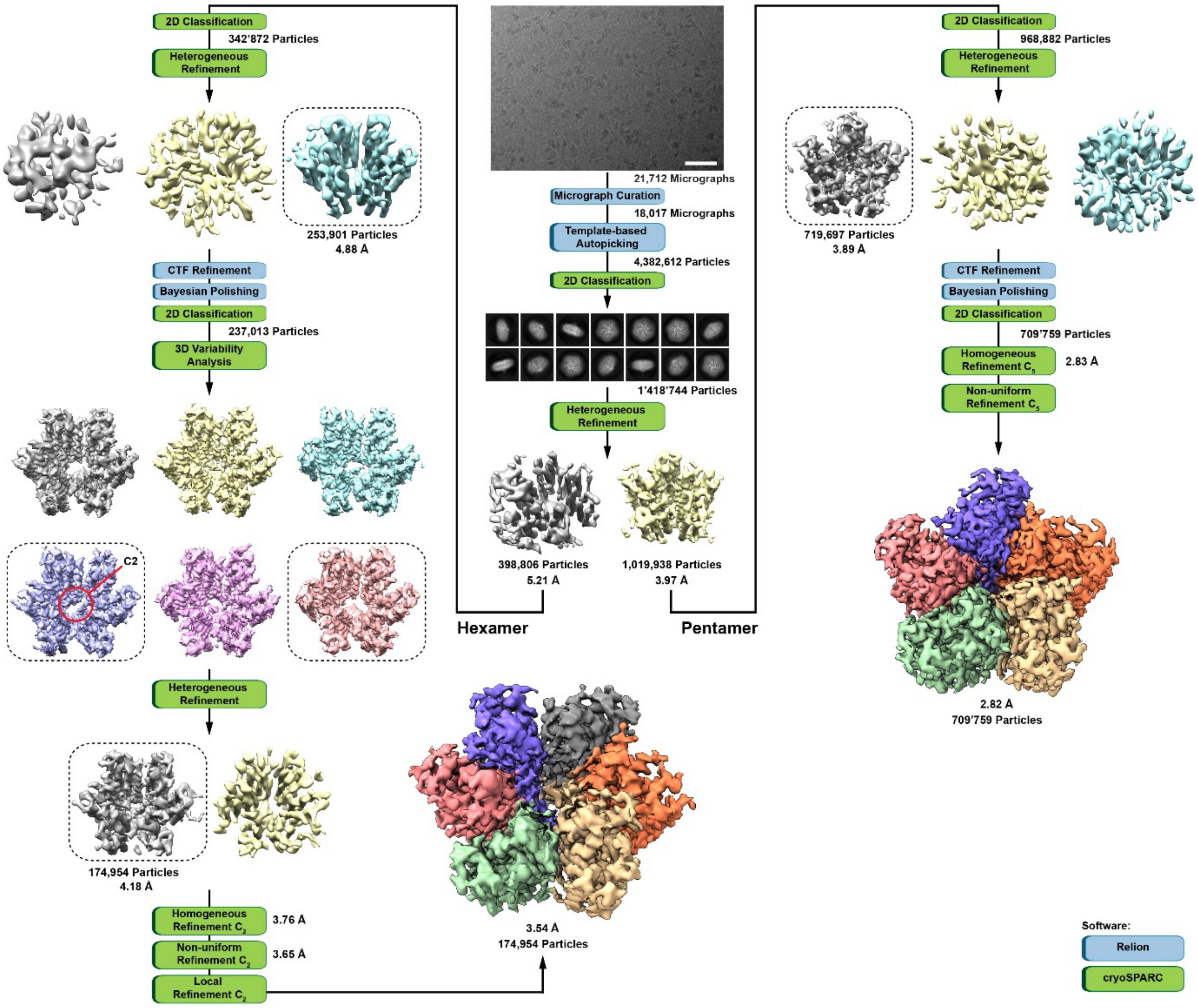

Supplementary Fig. 2: Cryo-EM data processing and analysis of GPR-A18L. The flowchart illustrates the single particle cryo-EM data processing workflow starting from template-based particle autopicking of motion-corrected micrographs. Particles were curated by several rounds of 2D classification and a heterogeneous refinement, which allowed separation of hexameric and pentameric particles. The two particle populations were further processed as shown, with selected classes indicated by dashed boxes. The colour-coding refers to the software packages used for the individual steps. The scale bar in the electron micrograph indicates 500 Å and the box size of the 2D classes is 180 Å.

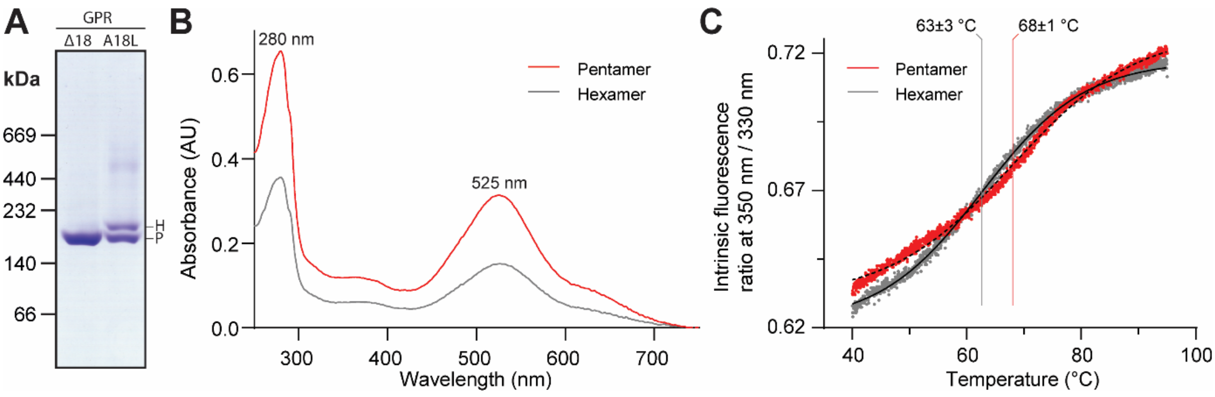

Supplementary Fig. 3: Blue native-polyacrylamide gel electrophoresis (BN-PAGE), UV-Vis spectroscopy and nano differential scanning fluorimetry (nanoDSF) of isolated GPR oligomers. (A) BN-PAGE of GPR without N-terminal signal sequence ($GPR_{\Delta18}$; *left lane*) and GPR-A18L variant (*right lane*) run on a native 4-16% Bis-Tris/polyacrylamide gel. $GPR_{\Delta18}$ runs as a single band corresponding to the pentamer. GPR-A18L appears as two bands corresponding to the pentameric (P) and hexameric (H) forms, with the pentamer being significantly more abundant. $GPR_{\Delta18}$ and GPR-A18L pentamers migrate similarly in the gel. (B) UV-Vis absorption spectra of GPR-A18L pentamers and hexamers isolated from BN-PAGE. The two oligomers exhibit similar absorption maxima of their retinal Schiff base at about 525 nm. The two displayed absorption spectra are representative from spectra of three independent oligomer isolations. (C) NanoDSF of isolated GPR-A18L pentamers and hexamers. Data from representative experiments using pentamer (red) and hexamer (grey) samples are shown. Inflection temperatures (T_i) were calculated from nonlinear regression fits and are indicated with standard deviations.

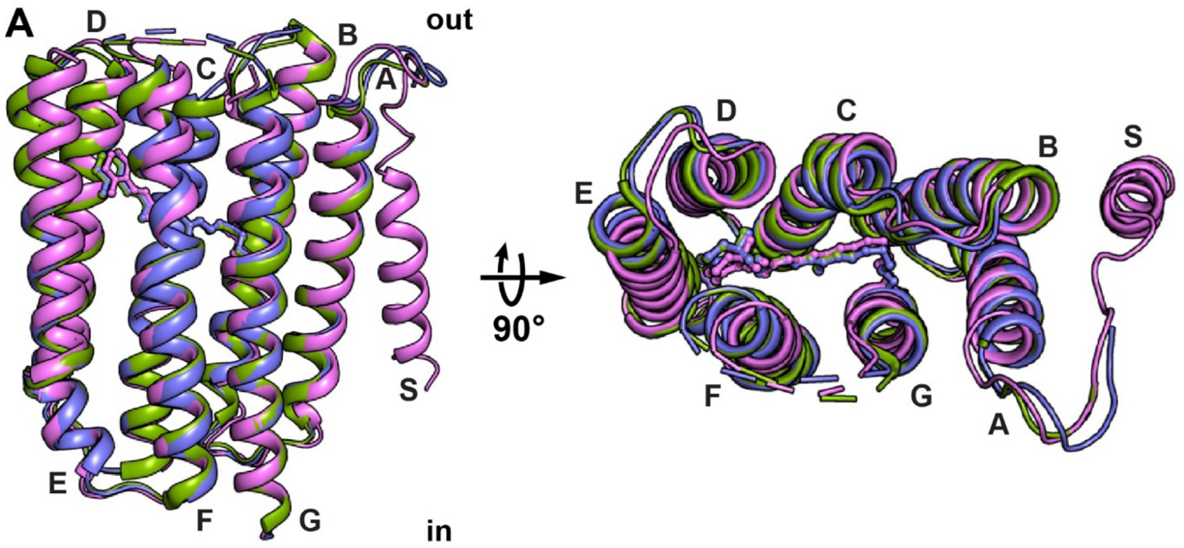

B

**1**	0					
**2**	0.914	0				
**3**	0.903	0.219	0			
**4**	0.014	0.877	0.899	0		
**5**	0.912	0.006	0.216	0.878	0	
**6**	0.908	0.214	0.009	0.902	0.211	0
**Protomer**	**1**	**2**	**3**	**4**	**5**	**6**

C

**1**	1.041	1.041	1.048	1.040	1.045
**2**	0.427	0.422	0.427	0.424	0.425
**3**	0.385	0.375	0.378	0.377	0.376
**4**	1.008	1.023	1.030	1.016	1.033
**5**	0.425	0.421	0.425	0.423	0.423
**6**	0.383	0.376	0.377	0.374	0.373
**Protomer**	**1**	**2**	**3**	**4**	**5**

Supplementary Fig. 4: Comparison of protomer structures in pentameric and hexameric GPR-A18L. (A) Structural superposition of GPR-A18L hexamer protomer 1 (magenta) and protomer 2 (blue), and pentamer protomer 1 (green). The top view is seen from the extracellular side. Transmembrane α-helices are labelled A-G and the signal peptide S. Structural alignment among GPR-A18L hexamer protomers (B) and compared to GPR-A18L pentamer protomers (C) expressed as overall root-mean-square deviation (RMSD) in Å.

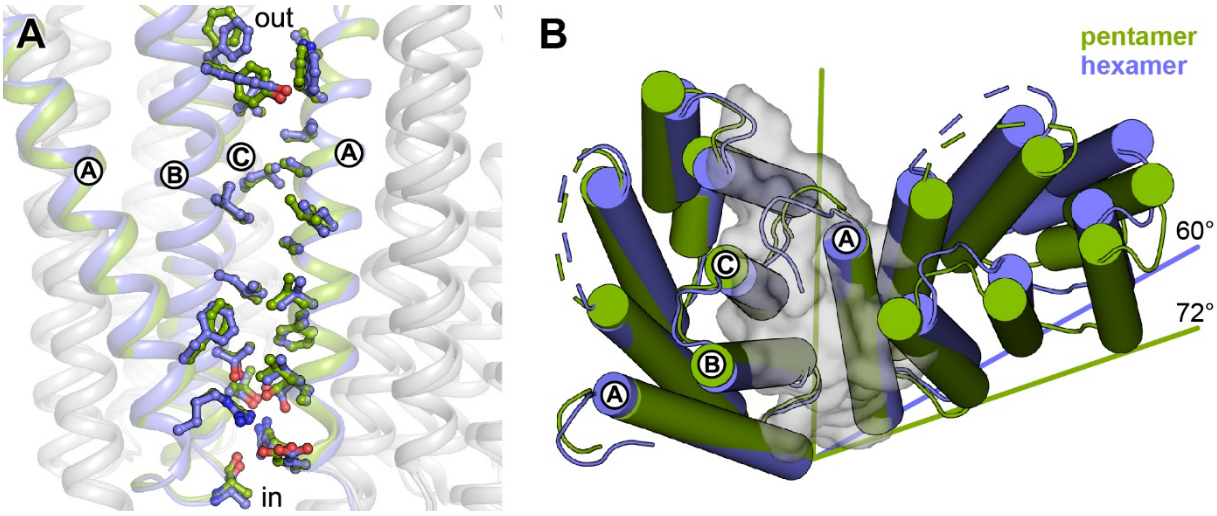

Supplementary Fig. 5: Oligomerisation interfaces in the GPR-A18L pentamer and hexamer. Comparison of interactions between neighbouring protomers 2 and 3 in GPR-A18L hexamer (blue) and pentamer (green) without involvement of the signal peptide. Transmembrane α-helices with residues participating in the interactions are highlighted and labelled. (B) Illustration of the different interface angles adopted by the protomers to form either a pentamer (green) or a hexamer (blue). The oligomerisation interface is represented by a grey volume.

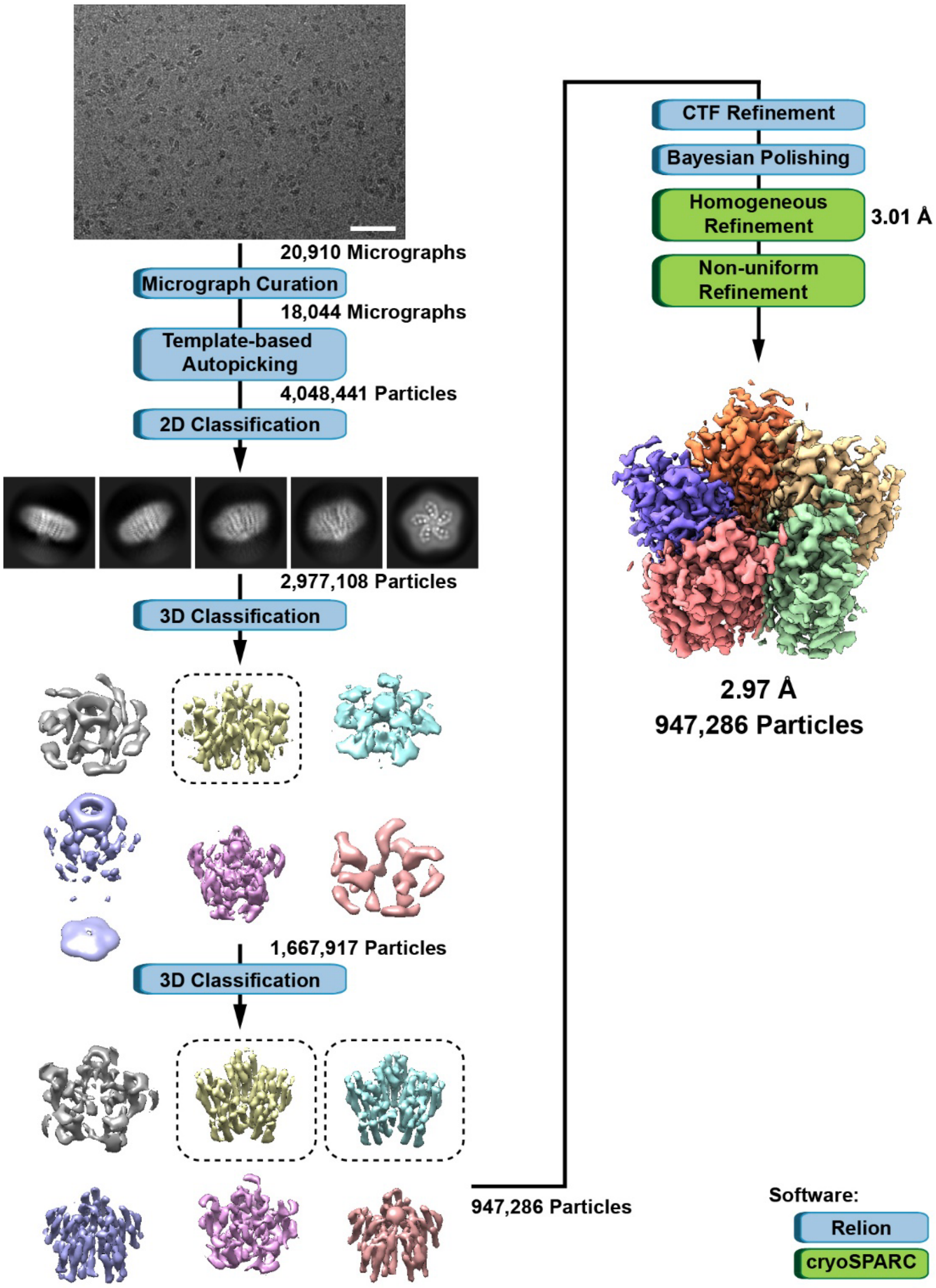

Supplementary Fig. 6: Cryo-EM data processing and analysis of proteoopsin. The flowchart illustrates the single particle cryo-EM data processing workflow starting from template-based particle autopicking of motion-corrected micrographs. Particles were curated by several rounds of 2D and 3D classification. Classes selected for further processing are indicated by dashed boxes. The colour-coding refers to the software packages used for individual steps. The scale bar in the electron micrograph indicates 500 Å and the box size of 2D classes is 180 Å.

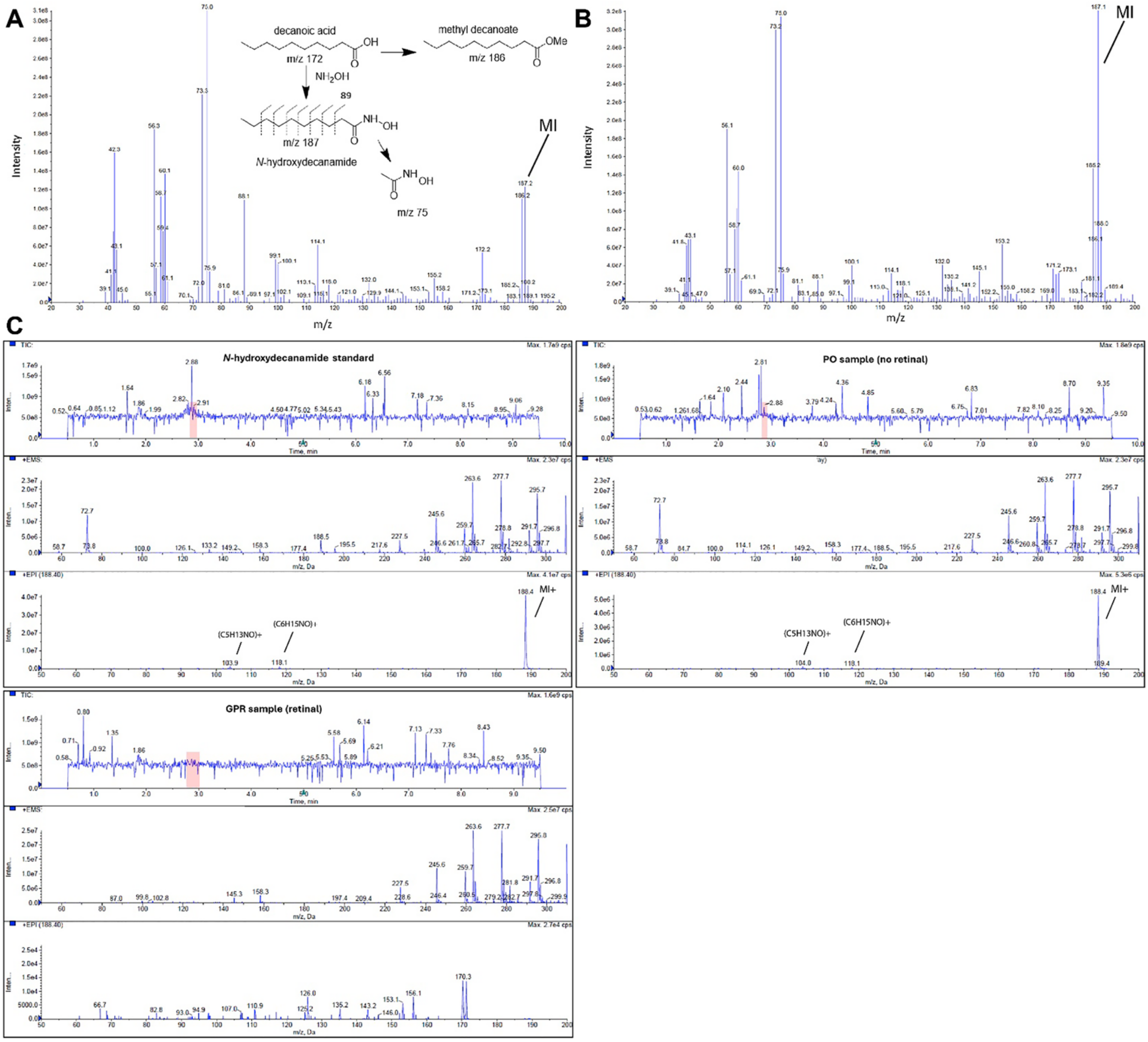

Supplementary Fig. 7: Representative direct injection quadrupole mass filter (+Q1) scans of (A) hydroxylamine (NH_2OH)-extracted decanoic acid from PO and (B) reaction products of decanoic acid control (Sigma W236403, >99.5%) with hydroxylamine in methanol/acetone, yielding *N*-hydroxydecanamide and methyl decanoate. Inset displays structures corresponding to major molecular ion peaks and fragmentations. (C) Representative information dependent acquisition (IDA) LC-MS/MS full spectrum ion survey scans of *N*-hydroxydecanamide (Sigma ENA408616050, >95%) standard, hydroxylamine extracted PO (no retinal) and GPR (retinal) samples. Corresponding (red area in survey scan) electron momentum spectroscopy (EMS) and enhanced product ion (EPI) mass spectra are shown. *N*-hydroxydecanamide (retention time 2.88 min) shows specific ions at 188 *m/z*, 118 *m/z* and 104 *m/z*. (corresponding .wiff files are available, see *Data availability* statement). Experiments were performed in more than three independent samples and extractions.

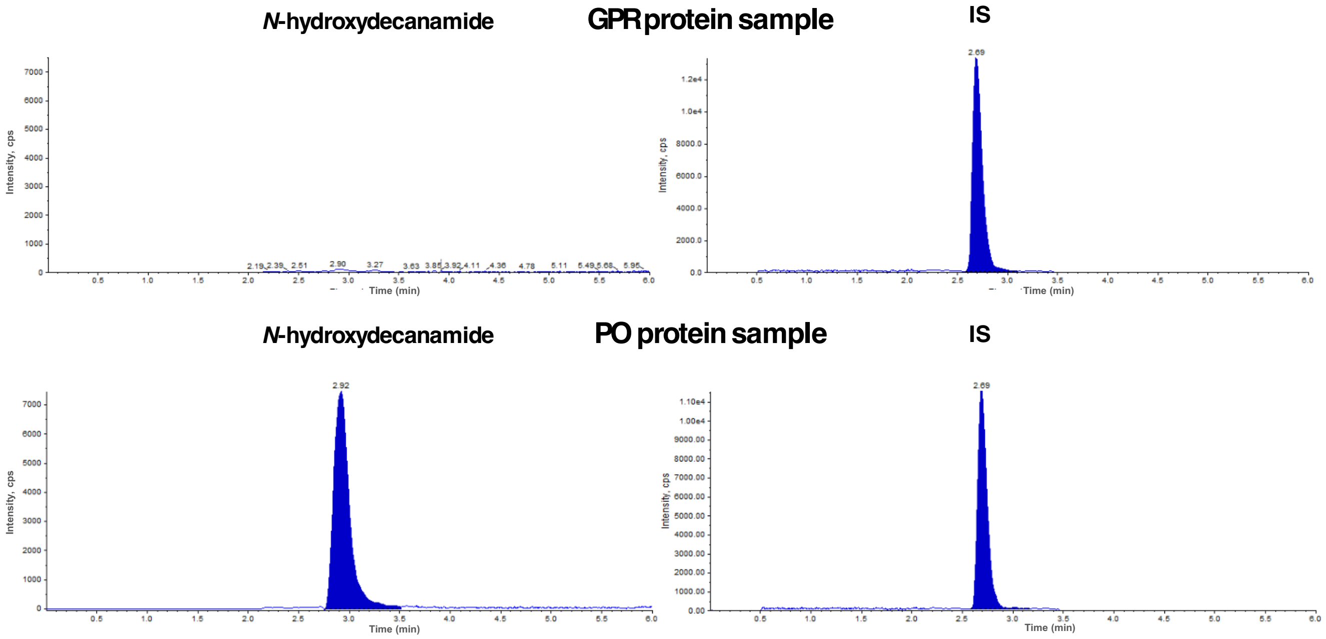

Supplementary Fig. 8: Representative chromatograms of LC-ESI-MS/MS experiments performed on a Sciex5500 MS device using an MRM method for *N*-hydroxydecanamide (syn. decanohydroxamic acid) (precursor ion 188 *m/z*, and product ions 104 *m/z* and 118 *m/z* in positive polarity) from comparable protein amounts extracted with hydroxylamine, which reacts with decanoic acid to *N*-hydroxydecanamide. Extraction from PO yielded 0.7 ng/mL *N*-hydroxydecanamide on column. Palmitoylethanolamide-d4 (precursor ion 305 *m/z* and product ion 62 *m/z* in positive polarity) was used as internal standard (IS) for the extractions. Experiments were performed in more than three independent samples and extractions.

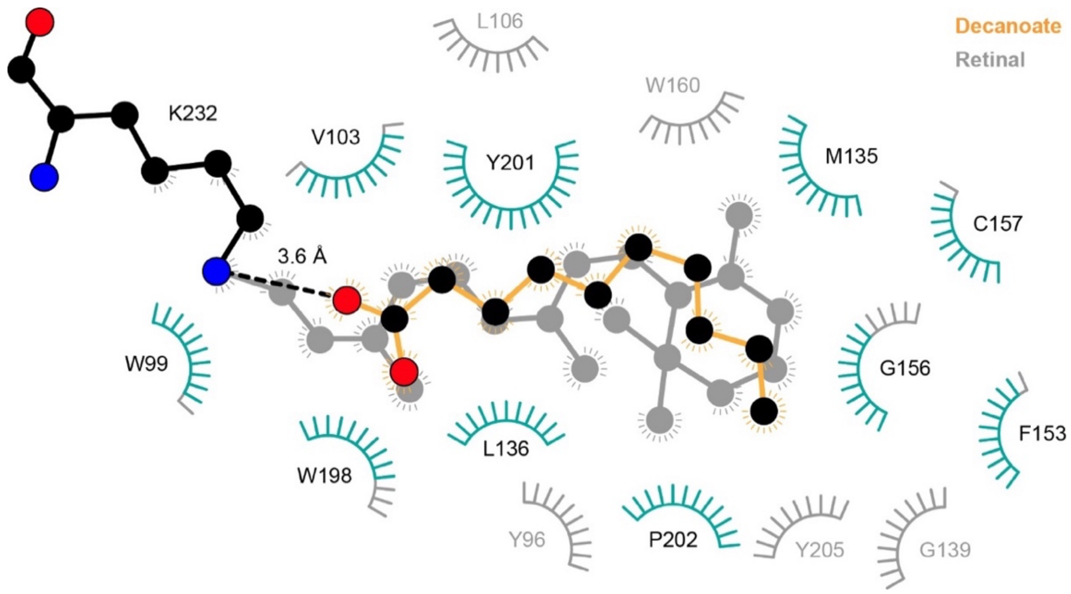

Supplementary Fig. 9: Protein-ligand interaction diagram for retinal-bound GPR and decanoate-bound PO. Interactions between decanoate (orange) and PO are overlaid on top of retinal (grey) and GPR. Side chains only interacting with retinal are coloured in grey, whereas side chains interacting with both ligands are shown in turquoise. Polar interactions are represented with dashed lines and distances are indicated. The figure was prepared using the LigPlot+ software (EMBL-EBI)[3].

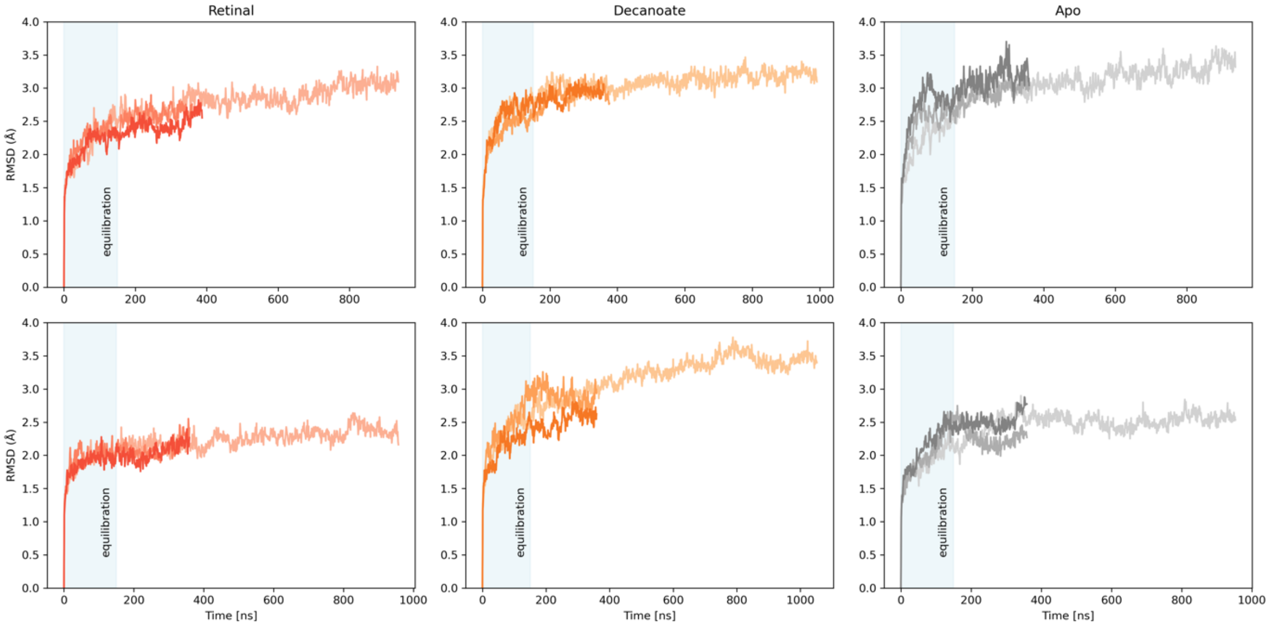

Supplementary Fig. 10: Root-mean-square deviation (RMSD) across simulations. The RMSD of all three replicates is shown in shades of red, orange and gray of molecular dynamics (MD) simulations of retinal- or decanoate-bound, and ligand-free proteoopsin. Simulations were seeded from lipid-embedded structures of proteoopsin (*top*) or green-light absorbing proteorhodopsin (PDB ID: 7B03) (*bottom*). The equilibration phase is highlighted with a light blue box.

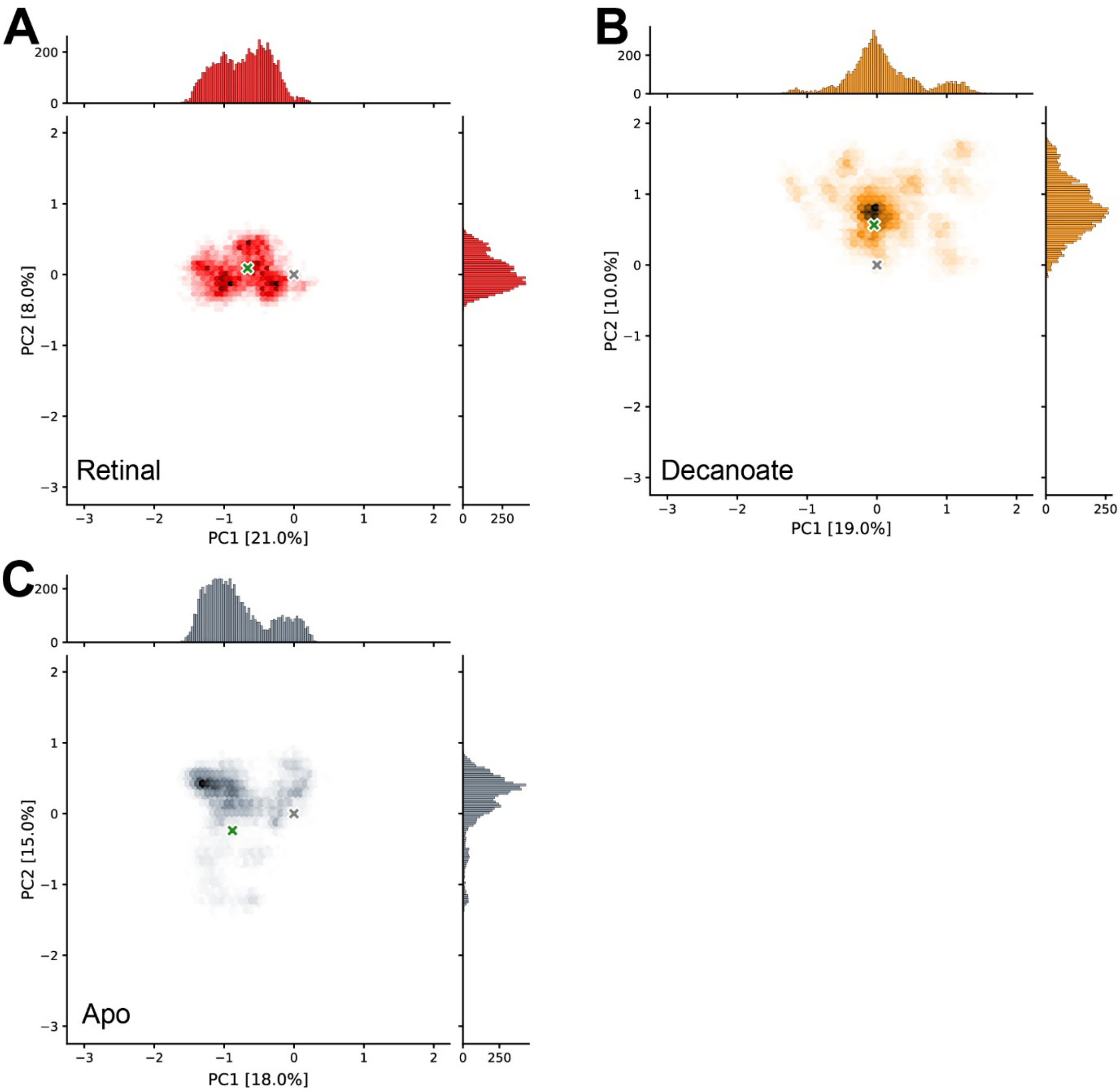

Supplementary Fig. 11: Principal Component (PC) analysis depicting the dynamic landscapes of molecular dynamics (MD) simulations of retinal- or decanoate-bound, and ligand-free proteoopsin. Simulations were seeded from lipid-embedded structures of proteoopsin (grey cross) or green-light absorbing proteorhodopsin (PDB ID: 7B03; green cross) bound to all-*trans* retinal by Schiff base (A), bound non-covalently to decanoate (B) or without ligand (C). The conformational densities are represented by hexagons, with increasing colour intensity reflecting the number of states in each bin. Marginal distributions for each PC are displayed on the respective axes.

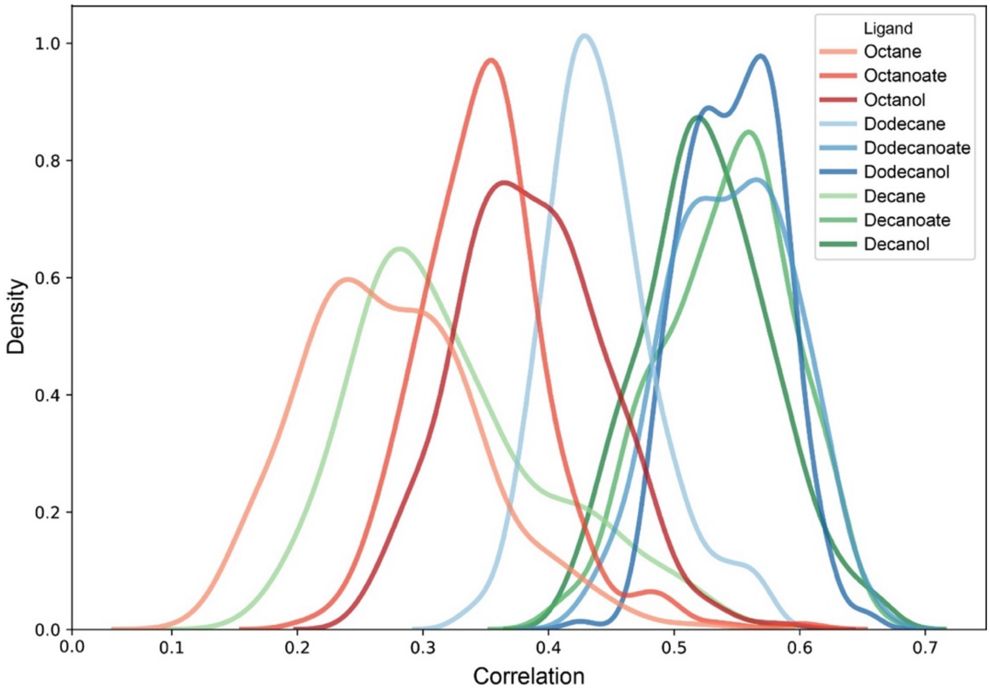

Supplementary Fig. 12: Correlation between computed and experimental densities for potential proteoopsin (PO) ligands. Molecular dynamics simulations of PO bound to different molecules were performed to assess their potential as substitutes for decanoate. The correlation of the computed densities for each ligand was then compared to the experimental cryo-EM density of decanoate.

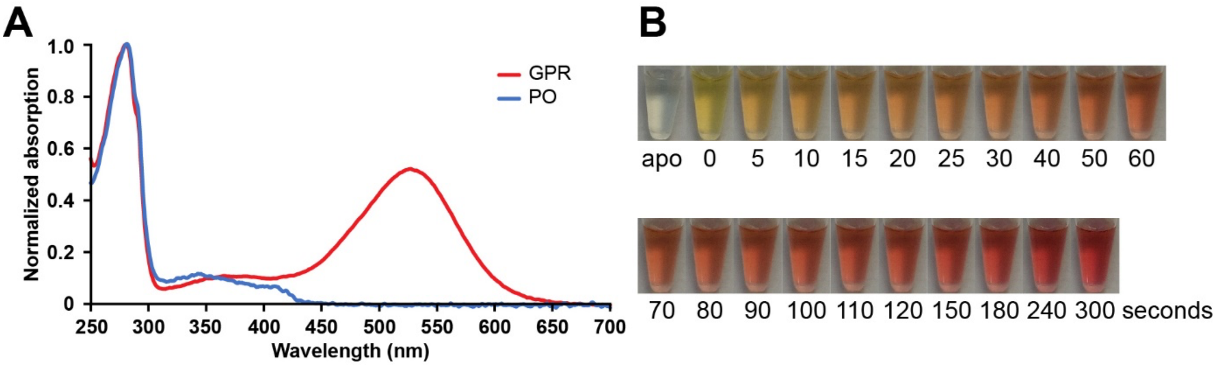

Supplementary Fig. 13: Spectral and visual observation of chromophore formation. (A) UV-Vis spectra of GPR (red), with the distinct retinal Schiff base absorption maximum at 525 nm, and PO (blue). (B) Time course (in seconds) of retinal Schiff base formation upon addition of 200 μM all-*trans* retinal to 1 mg/mL detergent-solubilised PO.

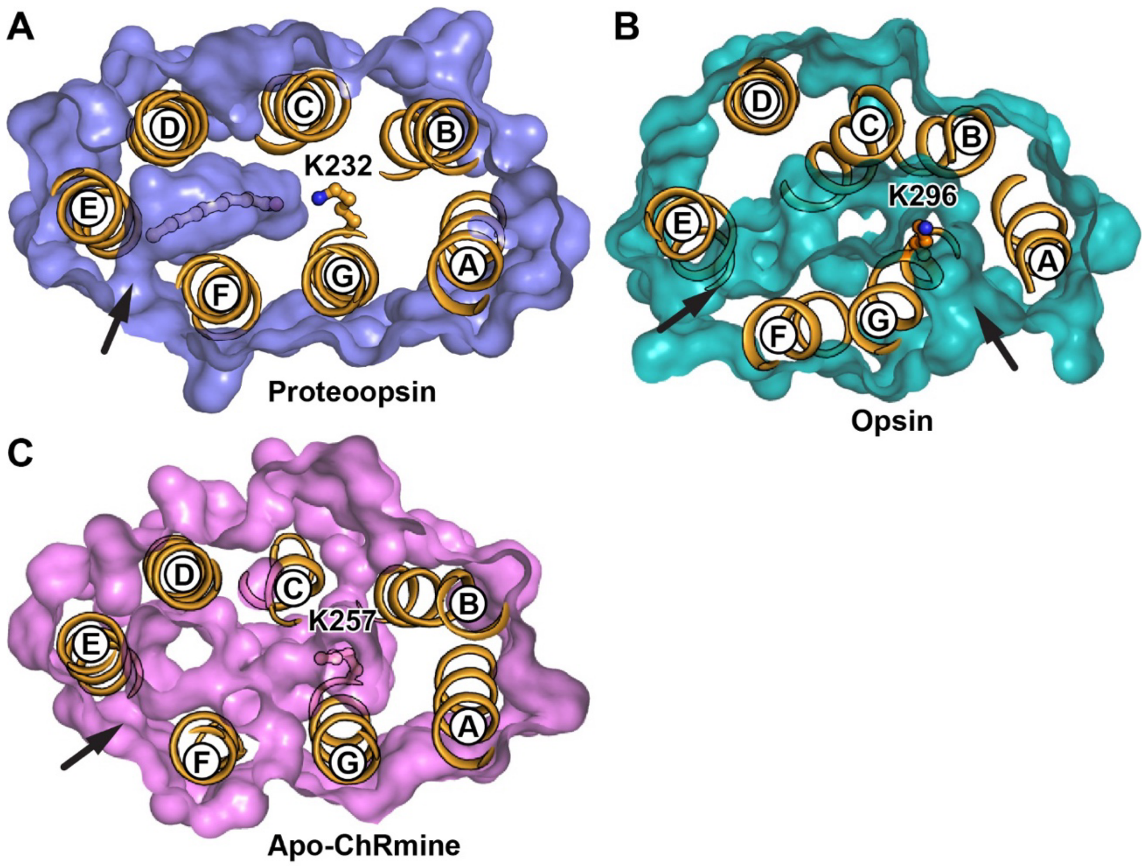

Supplementary Fig. 14: Potential access to the chromophore binding pocket in decanoate-bound PO and the ligand-free states of the G protein-coupled receptor opsin and the channelrhodopsin ChRmine. (A) Observed opening in PO cryo-EM structure between transmembrane α-helices (TMHs) E and F near the extracellular water interface (indicated with an arrow), connecting the ligand binding pocket to the lipid bilayer. (B) Potential ligand channel in opsin crystal structure (PDB ID: 3CAP) between TMHs E and F on one side and TMHs A and G on the other side (indicated with arrows). (C) An opening connecting to the binding pocket in the ligand-free structure of ChRmine (PDB ID: 7SHS) between TMHs E and F (indicated with an arrow). All top views are seen from the extracellular side. Decanoate and the lysine residues forming the retinal Schiff base (K232 in PO, K296 in opsin and K257 in apo-ChRmine) are displayed as ball-and-stick models, solvent accessible surfaces are represented in blue (PO), turquoise (opsin) or magenta (apo-ChRmine), and TMHs are labelled A-G.

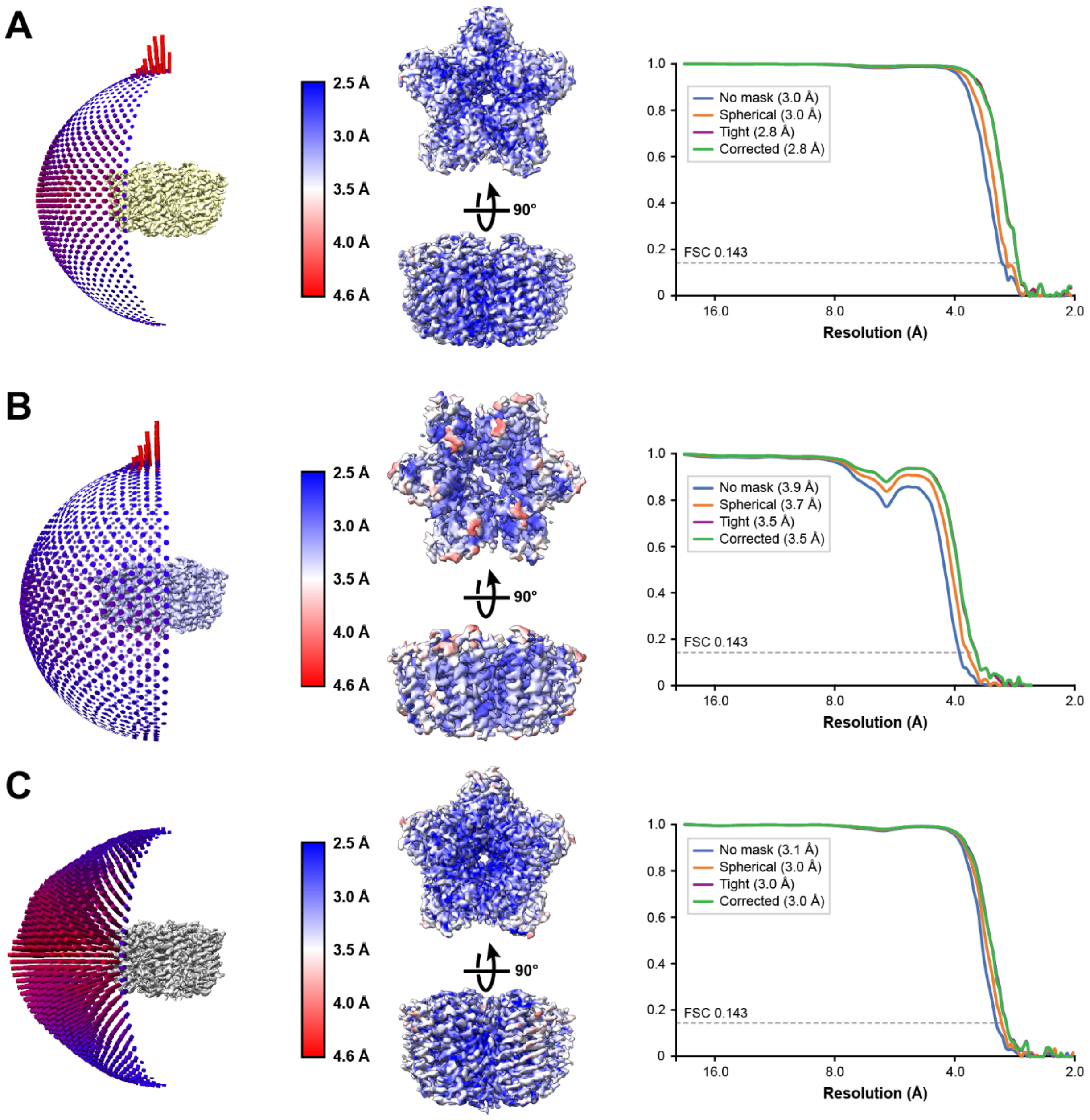

Supplementary Fig. 15: Analysis of GPR-A18L and PO cryo-EM maps. Three-dimensional distribution of particle Euler angles, representation of local resolution (calculated using MonoRes[4]) and Fourier shell correlation (FSC) curves for pentameric GPR-A18L (A), hexameric GPR-A18L (B) and pentameric PO (C).

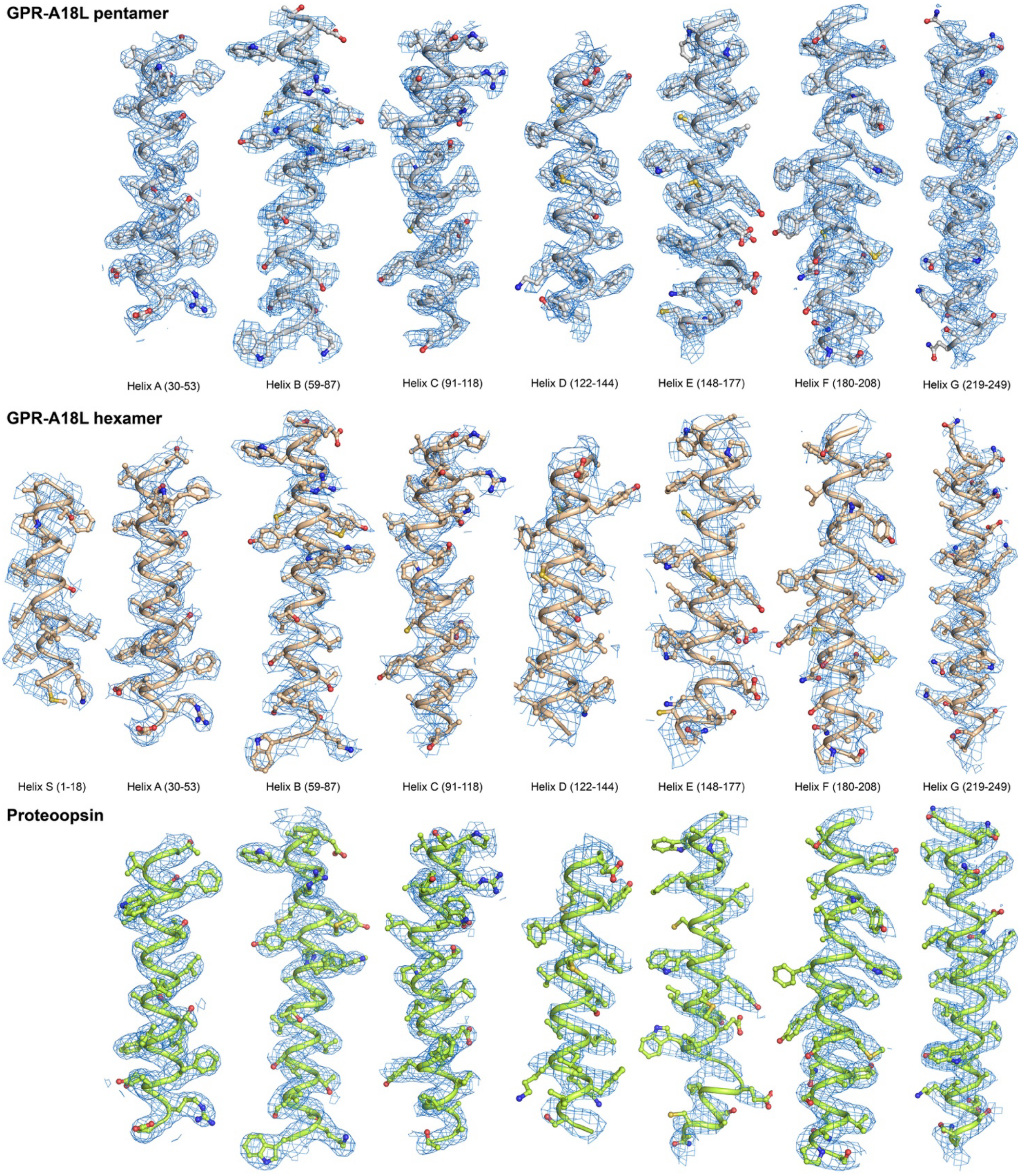

Supplementary Fig. 16: Cryo-EM map quality displayed for transmembrane α-helices of presented structures: GPR-A18L pentamer (PDB ID: 8CQC), GPR-A18L hexamer (PDB ID: 8CQD) and decanoate-bound proteoopsin (PDB ID: 8CNK).

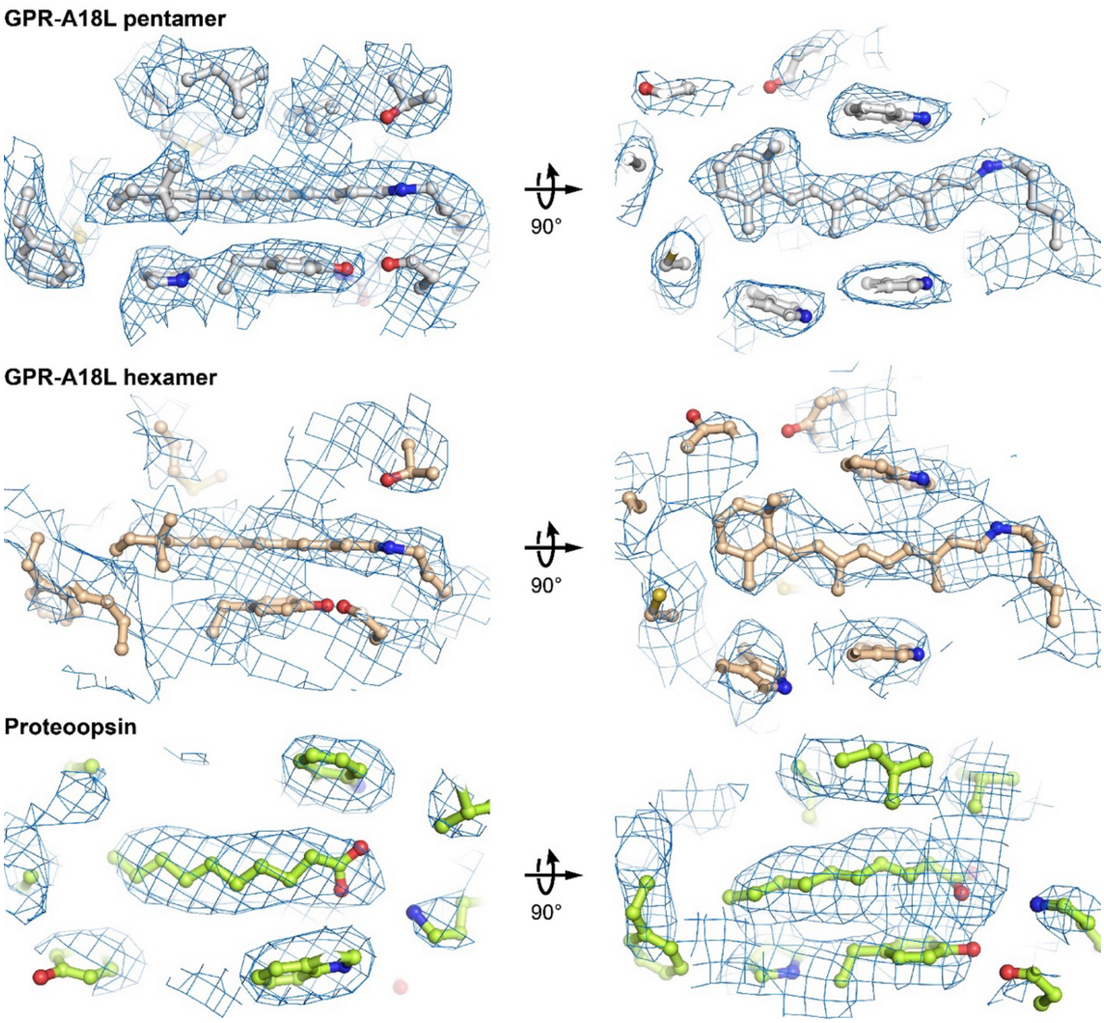

Supplementary Fig. 17: Cryo-EM map quality displayed for ligands and surrounding residues forming the ligand binding pocket of presented structures: retinal-bound GPR-A18L pentamer (PDB ID: 8CQC), retinal-bound GPR-A18L hexamer (PDB ID: 8CQD) and decanoate-bound proteoopsin (PDB ID: 8CNK).

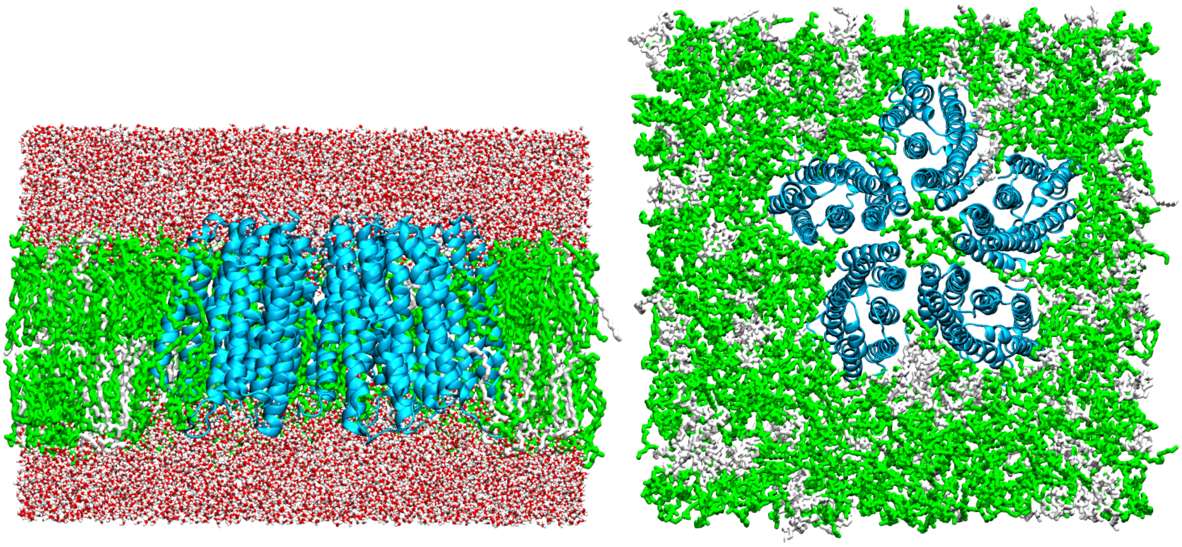

Supplementary Fig. 18: Representative atomic setup of the simulated systems. Side view (*left*) and top view (*right*) of a GPR pentamer embedded within bacterial mimetic membrane. The GPR is shown in cartoon representation (cyan) and lipid bilayer is composed of POPE (green) and POPG (white) in a 3:1 ratio. The side view additionally depicts the water layers surrounding the membrane.

Supplementary Tables

Supplementary Table 1: Cryo-EM data collection, refinement and validation statistics

	GPR-A18L		PO
**Data collection**			
Microscope	Titan Krios G3		Titan Krios G3
Camera	Gatan Quantum-K3		Gatan Quantum-K3
Voltage (kV)	300		300
Magnification	105,000x		130,000x
Defocus range (µm)	-0.7 to -1.7		-0.7 to -1.7
Movies recorded	21,712		20,910
Frames per movie	40		40
Total dose per movie ($e^-/Å^2$)	49.8		49.8
Pixel size (Å)	0.822		0.645
	**GPR-A18L Pentamer (8CQC)**	**GPR-A18L Hexamer (8CQD)**	**PO Pentamer (8CNK)**
**EM data processing**			
Initial particles picked	4,382,612		4,048,441
Particles used for refinement	709,759	237,013	947,286
Box size (Å)	180	220	180
Symmetry	C_5	C_2	C_5
Map resolution (Å)[a]	2.82	3.54	2.97
Map resolution range (Å)[b]	2.51 – 4.08	2.77 – 4.59	2.47 - 4.14
Map sharpening method	cryoSPARC	cryoSPARC	DeepEMhancer
**Model refinement and validation**			
Model composition			
Chains	5	6	5
Protein residues	1085	1364	1120
Ligands	5	6	5
Waters	30	0	0
RMSD			
Bond length (Å)	0.006	0.005	0.004
Angles (°)	0.684	0.566	0.678
Molprobity score[c]	1.34	1.12	1.10
Clash score	6.15	3.32	3.11
Rotamer outliers (%)	0.34	0	0
Ramachandran			
Favoured	98.03	98.13	99.55
Allowed	1.97	1.87	0.45
Outliers	0	0	0
Ramachandran Z-score	0.69	2.57	1.09
CaBLAM outliers (%)	0.34	0.46	1.85
EM-Ringer score[d]	2.25	0.67	2.61
Map CC (peak)[e]	0.59	0.52	0.61
Map CC (mask)[e]	0.79	0.73	0.80
Map CC (ligand)[e]	0.75	0.75	0.85

[a] Resolution determined by FSC with cut-off of 0.143.
[b] Resolution range defined by local resolution values at atom positions in Chimera.[5]
[c] Model statistics were calculated using MolProbity.[6]
[d] Calculated based on local fit of side chains to map according to Barad *et al.*[7]
[e] Real-space correlation coefficients of model-to-map fit as described in Afonine *et al.*[8]

Supplementary Table 2: System set-up for MD simulations

System with	Starting structure[a]	Dimensions [Å]	Number of atoms	Number of water molecules	Salt concentration NaCl [mM]	Lipid composition[b]	
						Number of POPE	Number of POPG
**Decanoate**	8CNK	142, 141, 91	166,152	28,330	150	377	124
**All-*trans* retinal**	8CNK	142, 141, 91	166,237	28,335	150	377	124
**No-ligand**	8CNK	142, 141, 91	166,007	28,335	150	377	124
**Decanoate**	7B03	142, 141, 91	165,973	28,352	150	377	124
**All-*trans* retinal**	7B03	142, 141, 91	166,058	28,357	150	377	124
**No-ligand**	7B03	142, 141, 91	165,828	28,357	150	377	124

[a] PDB ID code is indicated.

[b] Abbreviations of lipids: 1-palmitoyl-2-oleoyl phosphatidylethanolamine (POPE) and 1-palmitoyl-2-oleoyl phosphatidylglycerol (POPG).

Supplementary References

1. Hirschi, S., Kalbermatter, D., Ucurum, Z., Lemmin, T. & Fotiadis, D. Cryo-EM structure and dynamics of the green-light absorbing proteorhodopsin. *Nat. Commun.* **12**, 4107 (2021).

2. Hirschi, S., Kalbermatter, D., Ucurum, Z. & Fotiadis, D. Cryo-electron microscopic and X-ray crystallographic analysis of the light-driven proton pump proteorhodopsin reveals a pentameric assembly. *J. Struct. Biol. X* **4**, 100024 (2020).

3. Wallace, A. C., Laskowski, R. A. & Thornton, J. M. LIGPLOT: a program to generate schematic diagrams of protein-ligand interactions. *Protein Eng. Des. Sel.* **8**, 127–134 (1995).

4. Vilas, J. L. *et al.* MonoRes: automatic and accurate estimation of local resolution for electron microscopy maps. *Structure* **26**, 337–344 (2018).

5. Pettersen, E. F. *et al.* UCSF Chimera--a visualization system for exploratory research and analysis. *J. Comput. Chem.* **25**, 1605–1612 (2004).

6. Williams, C. J. *et al.* MolProbity: more and better reference data for improved all-atom structure validation. *Protein Sci.* **27**, 293–315 (2018).

7. Barad, B. A. *et al.* EMRinger: side chain–directed model and map validation for 3D cryo-electron microscopy. *Nat. Methods* **12**, 943–946 (2015).

8. Afonine, P. V. *et al.* New tools for the analysis and validation of cryo-EM maps and atomic models. *Acta Crystallogr. Sect. D Struct. Biol.* **74**, 814–840 (2018).
